# Galectin-1, a gene preferentially expressed at the tumor margin, promotes glioblastoma cell invasion

**DOI:** 10.1186/1476-4598-11-32

**Published:** 2012-05-14

**Authors:** L Gerard Toussaint, Allan E Nilson, Jennie M Goble, Karla V Ballman, C David James, Florence Lefranc, Robert Kiss, Joon H Uhm

**Affiliations:** 1 The Texas Brain and Spine Institute, 8441 St. Hwy 47, Suite 4300, Bryan, TX, 77807, USA; 2 Department of Neuroscience and Experimental Therapeutics, Texas A&M HSC College of Medicine, 2006 MREB, 8447 St. Hwy 47, Bryan, TX, 77807, USA; 3 Department of Neurology, Mayo Clinic, Rochester, MN, USA; 4 Division of Biomedical Statistics and Informatics, Mayo Clinic, Rochester, MN, USA; 5 Department of Neurological Surgery, UCSF, San Francisco, CA, USA; 6 Service de Neurochirurgie, Hôpital Erasme, Université Libre de Bruxelles, Brussels, Belgium; 7 Laboratoire de Toxicologie, Faculté de Pharmacie, Université Libre de Bruxelles, Brussels, Belgium

**Keywords:** Glioblastoma, Proliferation, Migration, Invasion, Galectin-1

## Abstract

**Background:**

High-grade gliomas, including glioblastomas (GBMs), are recalcitrant to local therapy in part because of their ability to invade the normal brain parenchyma surrounding these tumors. Animal models capable of recapitulating glioblastoma invasion may help identify mediators of this aggressive phenotype.

**Methods:**

Patient-derived glioblastoma lines have been propagated in our laboratories and orthotopically xenografted into the brains of immunocompromized mice. Invasive cells at the tumor periphery were isolated using laser capture microdissection. The mRNA expression profile of these cells was compared to expression at the tumor core, using normal mouse brain to control for host contamination. Galectin-1, a target identified by screening the resulting data, was stably over-expressed in the U87MG cell line. Sub-clones were assayed for attachment, proliferation, migration, invasion, and *in vivo* tumor phenotype.

**Results:**

Expression microarray data identified galectin-1 as the most potent marker (p-value 4.0 x 10^-8^) to identify GBM cells between tumor-brain interface as compared to the tumor core. Over-expression of galectin-1 enhanced migration and invasion *in vitro*. *In vivo*, tumors expressing high galectin-1 levels showed enhanced invasion and decreased host survival.

**Conclusions:**

In conclusion, cells at the margin of glioblastoma, in comparison to tumor core cells, have enhanced expression of mediators of invasion. Galectin-1 is likely one such mediator. Previous studies, along with the current one, have proven galectin-1 to be important in the migration and invasion of glioblastoma cells, in GBM neoangiogenesis, and also, potentially, in GBM immune privilege. Targeting this molecule may offer clinical improvement to the current standard of glioblastoma therapy, i.e. radiation, temozolomide, anti-angiogenic therapy, and vaccinotherapy.

## Backgound

In spite of recent advances in the treatment of patients with glioblastoma, the prognosis for those afflicted remains poor. Even when these tumors harbor a favorable gene methylation profile, the newest standard of care, including temozolomide as a chemotherapeutic [1], offers a median survival of less than two years [2]. Although extent of surgical resection is an important predictor of patient survival [3,4], local therapy for glioblastoma fails because microscopically invasive cells evade resection and eventually proliferate in spite of adjuvant chemoradiotherapy [5,6]. Controlling the invasive nature of this tumor may offer hope for more efficacious local therapy, improved quality of life, and perhaps better response to adjuvant therapies [5,6].

Numerous mediators of glioma cell migration and invasion have been identified, ranging from integrins [7,8] to focal adhesion proteins [9,10] and from upstream growth factor receptors [11] to effector metallo- [12,13] and serine- [14] proteases. Galectin-1 has also been identified as a key player in GBM cell migration [15]. The majority of these mediators were investigated in glioma because of their role in promoting cell migration in other cancers. Other mediators of GBM cell migration have been identified when investigators isolated invasive cells at the edge of glioblastomas and compared their gene expression to the cells collected from the tumor core [16-19]. In parallel, galectin-1 has been demonstrated to be more expressed in the invasive part of GBMs when compared to GBM core [20,21], while providing chemoresistance [22] and pro-angiogenic singals [23,24] to glioblastomas.

In this study, we investigated whether our patient-derived glioblastoma xenograft panel could reproduce the molecular signature of invasion seen in the human disease. Further, we hypothesized that expression of invasion mediators, and Galectin-1 specifically, would be elevated at the tumor-brain interface. By comparing microarray expression data from GBM cells at the tumor core to data from cells at the invasive edge, a list of the top 200 differentially expressed genes (by p-value) was subjected to a filtering algorithm. Galectin-1 was ranked at the top of this list. We thus took advantage of our own patient-derived glioblastoma xenograft model [25] in order to further decipher the roles of galectin-1 on GBM cell migration features. The system we have developed mitigates the effect of three important confounders from human samples. First, tissue is frozen within one minute of removal, ensuring high quality RNA. Second, the tumors grown typically do not reach sizes large enough to undergo necrosis at the tumor core, further assuring quality RNA from the tumor core and making the comparison of core to invasive edge more strictly reflective of the presence or absence of invasion (rather than hypoxia/necrosis versus invasion). Finally, the xenograft setting allows for harvesting of normal host brain from regions remote from the tumor, which serve as a control for possible contamination of samples microdissected at the tumor-brain interface. Galectin-1 was thus identified in this unsupervised method of analysis as a key marker of glioma invasion, while validating the novel filtering method (used to control for sample contamination) presented in this study.

## Materials and methods

### Human tumor line propagation

Patient-derived glioblastoma lines have been propagated in our laboratories as described previously [25,26]. Twenty-two separate human xenograft lines have been created and propagated. Of those, six were chosen for this study, representing varying known genetic alterations present in glioblastoma (e.g. EGFR amplification or mutation, PTEN deletion, p53 mutation, etc.). The use of human tissue to create these xenograft lines was performed in accordance with NIH guidelines and under approval of the Mayo Clinic Institutional Review Board (IRB protocol #802-04).

### Mouse orthopic xenografts

All nude mice (Hsd:Athymic Nude-Foxn1^nu^- Harlan Laboratories, Indianapolis, IN) used in this project were treated in compliance with NIH and institutional guidelines. These protocols were reviewed and approved by the Mayo Clinic Institutional Animal Care and Use Committee (IACUC protocols #84-05, #A279-09, #A688-11). Glioblastoma cells were injected intracranially into anesthetized mice following our laboratory protocol [25].

The U87MG (human glioblastoma cell line, ATCC Number: HTB-14™, ATCC, Manassas, VA) xenograft experiments were powered to have an 80% chance of detecting a change in xenograft survival of 10 days with a p-value of 0.05. Ten animals were needed per comparison group. Following injection, the mice were observed at least once daily until onset of neurological symptoms. At that time, animals were euthanized and their brains removed and either frozen using Optimal Cutting Temperature (OCT) compound (Electron Microscopy Sciences, Fort Washington, PA) in cryomolds placed atop dry ice, or fixed in 10% buffered formalin and paraffin embedded for histopathological analysis.

The symptom-free survival of nude mice harboring U87MG parental versus transfected xenografts was compared. Kaplan-Meyer survival curves were generated and comparisons made by Log-Rank testing.

### Laser capture microdissection

**Figure 1:**
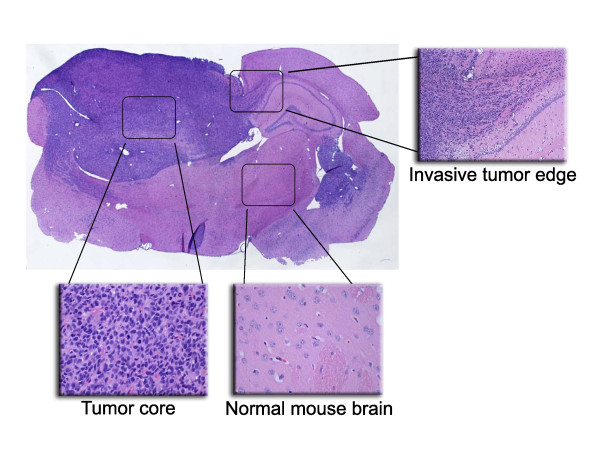
**Three geographic tumor regions targeted for RNA isolation.** Using laser-capture microdissection, tumor cells were isolated from the cell-dense tumor core and the tumor-brain interface. For comparison, cells were also collected from unaffected areas of the normal mouse brain. Modified with author permission [25].

Laser capture microdissection was used to collect samples from frozen, stained (HistoGene, Arcturus, Mountain View, CA), and dehydrated slides using previously published methods [27] on a PixCell IIe Laser Capture Microscope (Arcturus, Mountain View, CA). From the tumor cores, 3000 to 5500 cells per slide were collected, while the tumor edge allowed for collection of 300–900 cells per slide. Approximately 1000 to 2000 cells were collected from each sample of normal mouse brain (Figure 1).

### RNA isolation, quantitation, and quality control

The CapSure HS (Arcturus, Mountain View, CA) caps holding microdissected cells were coupled to their corresponding extraction buffer reservoirs. The column-based PicoPure RNA isolation kit (Arcturus, Mountain View, CA) was used to extract RNA from collected cells per manufacturer instructions.

The extraction buffer from eight slides per animal was pooled onto three separate columns (tumor core, tumor periphery, and normal brain) and totalRNA was eluted at the final step into a final volume of 11 microliters. One microliter of each eluted RNA sample was used for quantitation with the RiboGreen (Molecular Probes, Eugene, OR) assay kit. These total RNA samples were analyzed for integrity by obtaining electropherograms on an Agilent 2100 Bioanalyzer chip. Samples of acceptable quality based on RNA integrity number (2100 Expert software, Agilent Technologies, Inc. Palo Alto, CA) and plot profile (18 S and 28 S peaks with no degradation peaks) were processed for eventual chip hybridization as follows.

### RNA amplification and chip hybridization

Each sample was processed at the Mayo Clinic Genomic Center Microarray Shared Resource strictly according to Affymetrix small-sample protocol recommendations, and GeneChip hybridization followed our standard protocol [28]. After washes, arrays were scanned using the GeneChip Scanner 3000 (Affymetrix, Santa Clara, CA). Light intensity data from all spots on each chip were recorded as .cel files, stored on an internal server, and written to a digital variable disc (DVD).

### Data normalization and filtering

Raw data from chip hybridization experiments were normalized across chips and across probesets using a fast linear Loess routine [29], known as Fastlo. This normalization routine, in some respects similar to GC-RMA, does not subtract mismatch probe hybridization intensity from perfect match data. As a result, the reproducibility of the data is more robust (i.e., smaller p-values), while the fold-change ratios are not as extreme as with routines that use mismatch probe data in normalization.

By comparing expression data from cells at the tumor core to data from cells at the invasive edge, a list of the top 200 differentially expressed genes (by p-value) was subjected to a filtering algorithm. This algorithm was designed to minimize the effect of potential contamination of the edge samples with normal mouse brain cells. Relative expression values for each gene from tumor core, tumor edge, and normal mouse brain samples were compared. Genes of interest were identified that met three criteria: a) low expression at tumor core; b) relatively increased expression at tumor periphery; and c) lower signal (relative to tumor periphery) generated from surrounding rodent brain parenchyma. This profile obviates the concern that genes upregulated at the tumor edge might be identified because of cross-hybridization from mouse cells contaminating these edge samples. Conversely, genes were considered of interest if the opposite were true: lowest relative expression value at the tumor edge compared to tumor core and normal brain. The genes meeting this ideal profile were ranked by p-value (between core and edge).

### Immunohistochemistry

Formalin-fixed paraffin-embedded sections were stained using an ABC kit (Vector Laboratories, Burlingame, CA) after steam antigen retrieval. Primary antibodies included a mouse monoclonal anti-galectin-1 antibody (Research Diagnostics, INC, New Jersey) along with a human-specific mouse monoclonal anti-vimentin antibody (Dakocytomation, Trappes, France). After incubation with the provided goat anti-mouse secondary antibody, staining developed with NovaRed Developing Reagent (Vector Laboratories, Burlingame, CA). Sections were permanently mounted with Vectamount (Vector Laboratories, Burlingame, CA). Images were captured electronically.

### Vector creation

A cDNA clone of human galectin-1 was obtained from ATCC in the pOTB7 vector (ATCC #MGC-1818) and amplified using primers harboring BglII (forward) and SalI (reverse) sites:

· forward - CGGATCAGATCTTGTAAAACGACGGCCAGTAAC,

· reverse - CATCGTTGTCGACATGGGCTGGCTGATTTCAGTC),

Both this PCR product and the pIRES2-acGFP1 vector (Clontech, Mountain View, CA) were cut, using these restriction sites, subsequently gel purified, and finally ligated together (Roche Rapid Ligation Kit, Roche). The resulting pIRES2-Gal1-acGFP1 vector was transformed into Top10 cells (Invitrogen, Eugene, OR) for amplification. In parallel, a PCR reaction with primers harboring the same useful restriction sites (BglII forward and SalI reverse) was used to duplicate the acGFP1 sequence:

· forward – CGGATCAGATCTATGGTGAGCAAGGGCGCCGAG,

· reverse - CATCGTTGTCGACGCGGCCGCTCACTTGTAC.

This sequence was inserted into the multiple cloning site of the same vector pIRES2-acGFP1 to create one vector harboring two copies of acGFP. This circumvented a common problem with IRES vectors – inability to transcribe the transcript following the IRES sequence if the first MCS is empty. The new pIRES2-acGFP1-acGFP1 vector was used as a control for the pIRES2-Gal1-acGFP1 construct. Both vectors were sequenced through their multiple cloning sites to ensure no PCR-induced mutations were present.

### Transfection and stable clone generation

The U87MG human glioma cell line was kept in tissue culture in DMEM (Cellgro Mediatech, Inc.), with 10% fetal bovine serum, and penicillin/streptomycin. For transfection, 2.5x10^6^ cells were plated overnight on a 100 mm round dish. Cells were transfected using 60μL of Superfect reagent and 10μg of DNA in 10% fetal bovine serum media. After incubation for 3 hours, the adhered cells were washed four times with PBS and the media was replaced with our standard incubation media. Geneticin (G418) selection began the day after transfection. When enough GFP-expressing cells were identified, single cell suspensions were sorted under sterile conditions using the GFP filters (488/530 nm excitation/emission) on a FACS Vantage Sorter (Beckton Dickinson Immunocytometry Systems, San Jose, CA). Cells were collected in 96-well plates at a setting of 2 cells / well, after attempts to collect 1 cell per well failed to produce any viable clones. The remainder of the cells were collected and cultured under selection as a mixed population of transfected cells. All transfected cells were maintained in G418-containing media during the entire experiment.

### Western blotting

Lysates of cell pellets were made in 2X SDS sample buffer (Invitrogen, Carlsbad, CA). Gels were run at 150 volts until adequate separation of the 35 kDa and 50 kDa bands (60–90 minutes) of the Rainbow molecular weight maker (Amersham, Piscataway, NJ). Proteins were transferred to nitrocellulose and these membranes were incubated with primary antibody for 60 minutes (anti-Gal1 from Research Diagnostics, Flanders, New Jersey, anti-beta actin from Sigma, St. Louis, Missouri). After washing and incubation with secondary antibody (Goat Anti-Mse IgG-HRP, Pierce, Rockford, IL), developing solution was added to the membrane (Supersignal West Femto Substrate, Pierce, Rockford, IL). The time of film exposure was adjusted for optimal signal to background ratio.

### In vitro proliferation assays

Parental, control, and galectin-1 transfected U87MG cells were plated at a density of 1000 cells/well, each in eight wells of multiple 96-well plates. An MTS assay (Promega, Madison, WI) was performed at six hours after plating and daily thereafter until well overgrowth, according to the manufacturer’s protocol. Eight wells containing media alone were used to normalize the absorbance values of the other wells. Average corrected absorbance was compared between transfectant and parental cells, using a t-test.

### ECM attachment assays

Each well of a 96-well plate was incubated with 50μL of a fibronectin (Invitrogen, Eugene, OR) solution at 5 ng/μL. Once coated, parental, control, and galectin-1 transfected U87MG cells were introduced at a density of 10,000cells/well with eight replicate wells per line. After four hours, the media in half the wells was changed carefully – using a pipetteman rather than suction aspiration. An MTT assay (Promega, Madison, WI) was performed another four hours thereafter according to manufacturer’s protocol. The plate was read the next day, measuring absorbance at 570 nm and a reference wavelength of 655 nm. Average corrected absorbance data were normalized to the mean corrected absorbance of the U87MG parental cells.

### Radial migration assays

A two-dimensional radial migration assay was performed as previously described [30,31]. In a blinded fashion, various U87Gal-1 clones were analyzed and compared to U87GFP controls and parental U87MG cells. Of each clone, 2500 cells were allowed to sediment through a cooled manifold onto laminin-coated cell culture wells (Creative Scientific Methods, Inc., Phoenix, AZ). The manifold and slide were incubated together overnight. After removal of the manifold in the morning, an initial photograph was made (t_0_) of the circular cell colony in each well using an inverted microscope (Axiovert; Carl Zeiss, Thornwood, NY). A follow-up photograph of each cell colony was made at 24 hours. Using image analysis software (Scion Image, Frederick, MD), the change in radius of each cell colony over 24 hours was calculated. Data for each cell line was averaged over 10 wells and compared between parental, control transfected, and galectin-1 transfected cells. A t-test was applied to compare means.

### In vitro invasion assay

A modified Boyden chamber assay was performed using known protocols [31]. One day after plating at uniform density, 150,000 cells of each clone or line tested were added to the top of rehydrated Matrigel Invasion Chambers with 8micron pores (Becton Dickinson, Bedford, MA). We used 10% fetal calf serum as the chemoattractant in the bottom chamber. After 16 hours, the non-invading cells were removed with cotton swabs. Those cells that had migrated to the lower side of the membrane were fixed and stained with hematoxylin. Stained membranes were removed from the Boyden inserts, inverted, and permanently mounted on glass slides using ProLong Anti-Fade (Invitrogen, Eugene, OR). The invading cells on quadruplicate membranes were quantitated by counting across a diameter of each membrane under 40X microscopic magnification. Mean data were normalized in each assay to the number of parental cells invading the membranes; t-tests were used to determine statistical significance.

## Results

### Identification of galectin-1 as a potential mediator of glioma invasion

The quantity of RNA obtained from various xenograft tumors was highly variable. The average amount isolated from cells at the tumor periphery was 56.7 ng (± 40.0 ng), from the tumor core, 221 ng (± 109 ng), and from the normal mouse brain, 73.6 ng (± 38.2 ng). Across all three regions, the average amount of RNA isolated per cell was 6.8 pg +/− 4.2 pg. In spite of this variability, the quality of the RNA was consistently high with a mean RNA integrity number of 8.13 ± 0.74.

**Figure 2:**
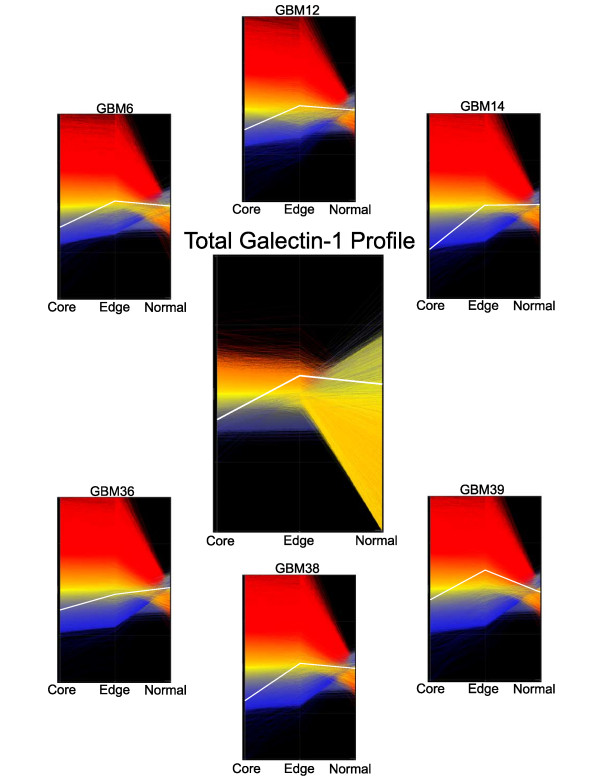
**The galectin-1 expression profile fits the criteria for a gene candidate that is relatively over-expressed at the tumor periphery.** These graphical representations of gene expression data compare the relative expression of galectin-1 from the core and edge of tumors to pooled data from normal mouse brain samples. (Graphics from GeneSpring®).

Expression microarray data suggested differential hybridization of RNA isolated from the tumor core and periphery for 12,796 separate probesets. The entire list of over 54,000 probesets was filtered as described for candidate genes meeting our criteria for an ideal expression profile. The resulting 8,969 probesets were ordered by p-value. The probeset encoded 216405_at on the Affymetix chip was at the top of this narrowed list, which hybridized to mRNA encoding the galectin-1 protein (p-value 4.0 × 10^-8^). The fold change calculated using our normalization routine was 2.04 in favor of the tumor-brain interface as compared to the tumor core. This is in comparison to a fold change of 4.31 when using GeneSpring, a commercially available program, with GC-RMA normalization (Silicon Genetics division of Agilent Technologies, Palo Alto, CA) (Figure 2).

### Protein-level confirmation of microarray data

**Figure 3:**
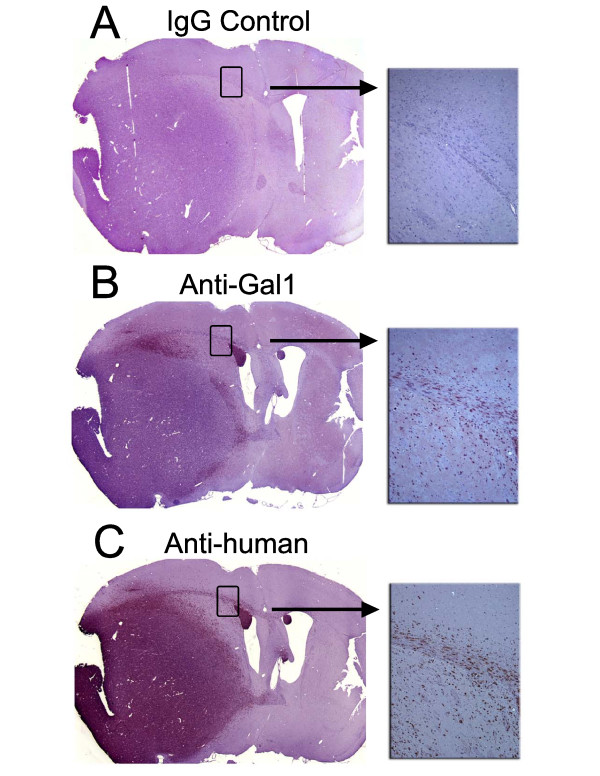
**Galectin-1 immunohistochemistry confirms preferential expression at the invasive edge of the glioblastoma xenograft.(A)** IgG control antibody **(B)** Galectin-1 staining **(C)** A human-specific vimentin antibody identifies all human cells in the field.

Paraffin sections of our patient-derived glioblastoma xenografts (15 of 22 lines) were stained for galectin-1 expression. Around half of the xenografts tested showed preferential staining at the tumor-brain interface (Figure 3). A few tumors stained in their entirety, and another subset lacked significant staining. The 2 to 4 fold change in galectin-1 mRNA expression at the tumor edge compared to core may not have been sufficient to cause a change in immunohistochemical staining. For those tumors where there was staining of cells invading surrounding host brain, we confirmed the human origin of these remote cells with a human-specific antibody stain (anti-vimentin). To control for the distinct isotypes of primary IgG, we stained parallel sections with a pooled IgG control. This protein-level confirmation of our microarray data gave us the impetus to pursue functional *in vitro* and *in vivo* assays with galectin-1 over-expressing GBM cells.

### Extracellular matrix attachment

**Figure 4:**
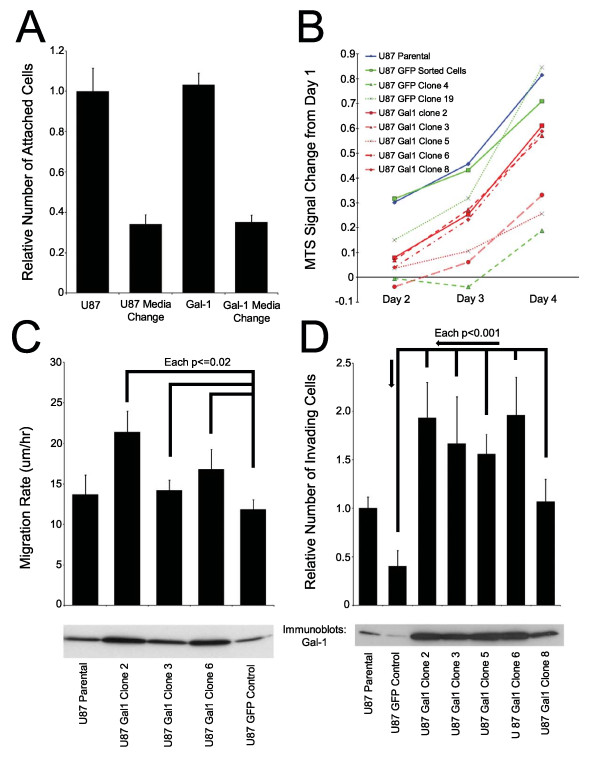
(**A**) Galectin-1 transfection does not alter U87MG attachment to fibronectin. Attachment to fibronectin-coated 96-well plates was quantitated with an MTT assay with and without a media change in the middle of the assay. Data were normalized to those of the parental line. One representative assay is presented. (**B**) **Galectin-1 transfected clones do not proliferate faster than controls.** The change in MTS signal from day 1 is plotted against time. (**C**) **Over-expression of galectin-1 promotes an increased rate of two-dimensional migration**. Galectin-1 transfected clones were compared to their GFP control counterparts. (Westerns controlled for loading by β-actin IB). (**D**) **Over-expression of galectin-1 promotes invasion.** All cell counts were normalized to the parental cell line data. (Westerns controlled for loading by β-actin IB)

The efficiency of attachment of U87MG cells to the extracellular matrix (ECM) was compared to galectin-1 transfectants. A population of GFP-sorted cells (the “Gal-1” bars in Figure 4A) was compared to its parental counterpart. The number of metabolically-active cells attached to fibronectin was no different between the two lines at eight hours. Changing the media at four hours reduced the number of cells left for labeling, but the effect was equal in both groups, suggesting a similar rate of attachment for the two populations.

### In vitro proliferation

Through cell sorting and selective clonal expansion, clones of galectin-1 and acGFP-only transfectants were created. To ensure that galectin-1 over-expression would not enhance proliferation of the U87MG line (and hence alter the interpretation of cell migration, cell invasion, and host survival assays) we measured MTS incorporation over time. Indeed, galectin-1 clones grew at a rate slightly, but not statistically significantly, lower than the U87MG parental cell line and sorted GFP-only cells. The growth rate of these cells rested between the rates of two acGFP-only control clones (Figure 4B).

### In vitro migration

The migratory rate of galectin-1 transfected cells was compared to parental U87MG and acGFP-only controls. Cells over-expressing galectin-1 proved to have migratory rates on laminin that were statistically significantly faster than cells transfected with acGFP alone. The migratory rates of the U87 galectin-1 clones tested paralleled the trend in galectin-1 expression seen in western blotting (Figure 4C).

### In vitro invasion

A Boyden chamber transwell invasion assay was used to assess the ability of various cell populations to invade through a membrane coated with Matrigel® and possessing 8μm pores. Overall, galectin-1 clones proved more efficient in invasion than their acGFP-only counterparts (Figure 4D). In addition, the relative invasion efficiency paralleled the level of galectin-1 expression (western blotting) for each clone tested (Figure 4D).

### U87MG GBM xenograft-bearing mice survival

**Figure 5:**
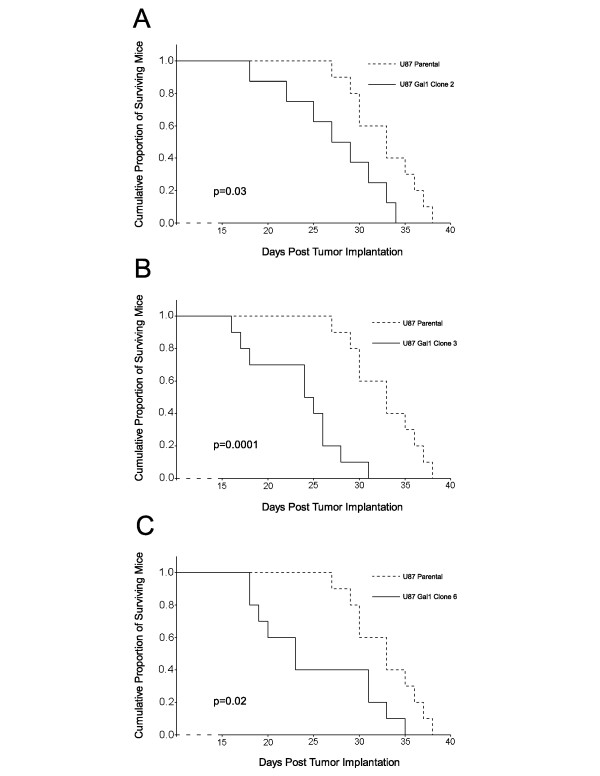
**Over-expression of galectin-1 in orthotopic xenografts decreases host survival.** Orthotopic xenografts of parental U87G and galectin-1 transfected cells were established in nude mice. Ten mice were assigned to each cell line, and each mouse received intracranial injection of 1 x 10^6^ cells under general anesthesia. At the onset of neurological symptoms, animals were sacrificed in accordance with the Mayo Clinic IACUC. Survival curves were generated from those animals (38 of 40) developing tumors (7 of 20 acGFP-only).

We assessed the survival of nude mice harboring xenograft brain tumors induced by a few of our U87 galectin-1 clones. Parental U87MG cells, along with galectin-1 and acGFP-only clones were injected into the right caudate/putamen complex of nude mice. Tumors over-expressing galectin-1 shortened survival of their hosts compared to their parental counterparts (Figure 5). A few animals (7/20) bearing tumors expressing acGFP alone eventually exhibited neurological symptoms. The examination of their brains suggested very little tumor burden, and significant hydrocephalus was seen in a few (3/7). The remainder of these did not suffer neurological decline, and the median survival was never reached.

### Xenograft tumor morphology

**Figure 6:**
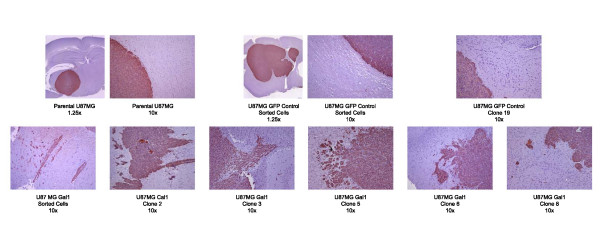
**Over-expression of galectin-1 in orthotopic xenografts alters the tumor phenotype.** Although the typical U87MG tumor xenograft is non-invasive, galectin-1 transfected U87MG clones formed tumors showing some invasion at the tumor-brain interface. Selected sections were stained using a human-specific anti-vimentin antibody.

All of our galectin-1 transfected clones generated tumors in nude mice. The pattern of growth was compared at the microscopic level to parental cells and those few tumors generated from acGFP-only clones. Paraffin sections were stained with human-specific anti-vimentin antibody to enhance the contrast between tumor cells (brown) and host brain (blue counterstain, Figure 6). The microscopic invasion shown for the galectin-1 transfectants was seen frequently, albeit limited to the local surrounding brain because U87MG tumors typically (see the Discussion) show little invasion. Galectin-1 immunohistochemical staining of the U87 galectin-1 clone xenografts were ubiquitously positive, without discernable overexpression at the tumor-brain interface.

## Discussion

This study illustrates a novel microarray data filtering algorithm that identified galectin-1 as a gene preferentially expressed at the glioblastoma-brain interface. The unique filtering of expression microarray data was made possible by the ample amount of normal mouse brain tissue available for dissection. In spite of species differences, cross-hybridization of mouse genetic material to human probes did prove to be a common occurrence. These data made it possible to control, rather stringently, for the potential contamination of tumor edge samples with mouse brain. Of course, there could still be possible contamination – reactive mouse astrocytes expressing unique genes that happen to cross-hybridize to the human chip could have altered the expression profile at the tumor-brain interface. Yet, this set of genes (those uniquely up- or down-regulated in the host’s reactive astrocytes around the xenograft tumor) must be orders of magnitude smaller that the set of all possible cross-hybridizing host genes.

The use of our animal model to identify mediators of glioma invasion has the potential pitfall of identifying artifacts of xenografting. That is, human glioma cells confronted with nude mouse brain rather than human brain may express genes specific to this setting. Two arguments can be made against this theory. First, there is no teleological reason for human cells to have an expression repertoire specific to mouse brain. In fact, there is an 81% concordance rate between our glioblastoma xenograft data for genes upregulated at the invasive tumor edge with p < 0.05 and those data derived from human samples (publication in preparation). This concordance compares favorably with the 70-85% concordance rate across microarray chip platforms when hybridizing the same sample RNA [32]. Additionally, our identification of galectin-1 as a mediator of glioma invasion has been corroborated previously as detailed below.

While previous studies from our group [22-24,33,34] and others [35,36] mainly focused on the effects provoked by lowering galectin-1 expression in GBM cell biology, the current study focuses of the reverse effects, e.g. the impact of over expressing galectin-1 in GBM cell biological behavior. The data from the present study, which rely on *in vitro* and *in vivo* assays of GBM cells stably transfected to over-express galectin-1, perfectly fit in with the previous studies mentioned above and highlight the importance of galectin-1 in the biologically aggressive behavior of experimental GBMs. While there was no enhancement of proliferation or change in attachment to fibronectin, galectin-1 upregulation induced more rapid two-dimensional migration and enhanced transwell invasion. Finally, mice harboring tumors grown from transfectant clones had shortened survival compared to those with parental-cell tumors. More strikingly, the most appropriate control xenografts, U87-GFP cells, formed tumors in only 7 of 20 implanted mice. U87 galectin-1 clone tumors showed enhanced local invasion compared to their parental counterparts. We did not observe, however, distant invasion in U87MG tumors over-expressing galectin-1. The U87MG model is in fact weakly invasive in the brains of immunocompromized mice [33,34], while it is associated with pronounced neoangiogenesis processes [37]. Further work (e.g. viral transduction) with our patient-derived xenograft lines is necessary to characterize completely the *in vivo* phenotypic alterations that accompany overexpression of galectin-1.

Our model system has identified galectin-1 as a major regulator of glioma invasion. Lending credence to the utility of short-passage human-derived xenografts in modeling tumor biology, the notion of galectin-1 promoting glioma invasion is well-supported by existing data from our lab and those of others. In fact, these over-expression data parallel our previous work, showing that galectin-1 added to the culture media markedly and specifically increased cell migration levels in human neoplastic astrocytes, and that these effects were related to striking modifications in the organization of the actin cytoskeleton and an increase in small GTPase RhoA expression [33]. Conversely, knocking down galectin-1 expression in U87MG GBM cells by stable transfection with antisense galectin-1 mRNA, the compliment to our current study, impairs motility and delays mortality after their intracranial grafting to nude mice [33]. In addition, stable transfection with antisense galectin-1 vector resulted in numerous gene expression alterations and modification of the actin stress fiber organization [34]. Altogether, the data from previous studies, completed by the current ones, highlight a major role for galectin-1 in GBM invasiveness.

The characteristic malignant phenotype of glioblastoma extends beyond aggressive invasion. This tumor develops resistance to chemo- and radio-therapy, it promotes neoangiogenesis, and it seems to benefit from immune privilege. Interestingly, galectin-1 may play a role in promoting each of these phenotypes. While galectin-1 may be secreted by GBM in relative hypoxia, it is markedly pro-angiogenic, and it is taken up by endothelial cells in a process that activates H-Ras signaling through Raf/Mek/Erk to promote endothelial proliferation [38-40]. The standard therapies for GBM – radiation and temozolamide both promote galectin-1 production, a possible survival reaction of the tumor [22,36]. Indeed, abrogating galectin-1 expression renders tumor cells more susceptible to temozolamide treatment [22,41]. Finally, galectin-1 induces apoptosis of activated T-cells [42-46], prevents host animals from mounting tumor vaccine-induced immunity [47], and may cooperate with TGF-beta in GBM-induced immunosuppression [48,49]. In sum, galectin-1 expression may inversely correlate with patient outcome as evidenced in previous work [33] and our current study (Additional file 1). With these important roles outside tumor invasion, galectin-1 proves to be a model molecule in our xenograft study. It was identified when searching for invasion mediators and understanding its biology leads to a greater understanding of glioblastoma in general.

In conclusion, the orthotopic glioblastoma xenograft model recapitulates not only the invasive phenotype, but also the regional expression profile reported in human samples of glioblastoma multiforme. The value of the model (i.e., abundant tissue, high-quality RNA, and fidelity to the human disease) makes it a resource for identification, as well as preclinical targeting, of novel mediators of glioma invasion. Galectin-1 was identified in this manner, and has proven *in vitro* and *in vivo* to be important in the migration and invasion of glioblastoma cells. Previous work suggests an even greater role of galectin-1 in GBM neoangiogenesis, chemo- and radio-resistence, and immune privilege. Targeting this molecule may offer clinical improvement to the current standard of glioblastoma therapy, i.e. radiation, temozolomide, anti-angiogenic therapy, and vaccinotherapy.

## Abbreviations

ATCC: American type culture collection; ECM: Extracellular matrix; GBM: Glioblastoma; LCM: Laser capture microscope; DMEM: Dulbecco’s minimum essential media; PBS: Phosphate buffered saline; OCT: Optimal cutting temperature® (freezing media); RIN: RNA integrity number; IVT: In-vitro transcription; GC-RMA: GeneChip robust multiarray averaging; GFP: Green fluorescent protein; acGFP: Aequorea coerulescens green fluorescent protein; FACS: Flow-assisted cell sorting; SDS: Sodium dodecyl sulfate; MTS: 3-(4,5-dimethylthiazol-2-yl)-5-(3-carboxymethoxyphenyl)-2-(4-sulfophenyl)-2 H-tetrazolium; MTT: 3-(4,5-Dimethylthiazol-2-yl)-2,5-diphenyltetrazolium bromide; CCD: Charge-coupled device.

## Misc

This work was supported by grants from the Sontag Foundation Distinguished Scientist Award (JHU), the NIH NRSA T32 NS 07494 (LGT) grant, the NIH LRP (LGT) grant, and the Mayo Clinic Clinician Investigator Training Program (LGT)

## Competing interests

None of the listed authors have competing interests related to the publication of this manscript.

## Authors’ contributions

LGT and JHU conceived of the study and designed the assays. LGT performed tumor xenografting, cell culture, and laser capture microdissection. LGT, FL, and RK wrote and edited the manuscript. AN designed and performed all DNA vector construction and sequencing. JG performed tissue culture, western blots, invasion assays, and immunohistochemistry. KB led the microarray statistical analysis. CDJ created the orthotopic xenograft model and provided guidance on its use in this project. All authors read and approved the final manuscript.

## Supplementary Material

Additional file1 **Figure S1.** Galectin-1 staining correlates with patient survival. Using a tissue microarray created at Mayo Clinic, we stained glioblastoma samples from 34 separate patients using immunohistochemistry for galectin-1. A survival analysis revealed a trend towards shorter survival in those patients harboring galectin-1 positive tumors. Click here for file
