# Supplementary material for: Galectin-1, a gene preferentially expressed at the tumor margin, promotes glioblastoma cell invasion
Source: Mol Cancer. 2012 May 14;11:32. doi: 10.1186/1476-4598-11-32 (PMC3407025; doi:10.1186/1476-4598-11-32)
Supplement: Additional file1 — Figure S1. Galectin-1 staining correlates with patient survival. Using a tissue microarray created at Mayo Clinic, we stained glioblastoma samples from 34 separate patients using immunohistochemistry for galectin-1. A survival analysis revealed a trend towards shorter survival in those patients harboring galectin-1 positive tumors. [file 1476-4598-11-32-S1.pdf]

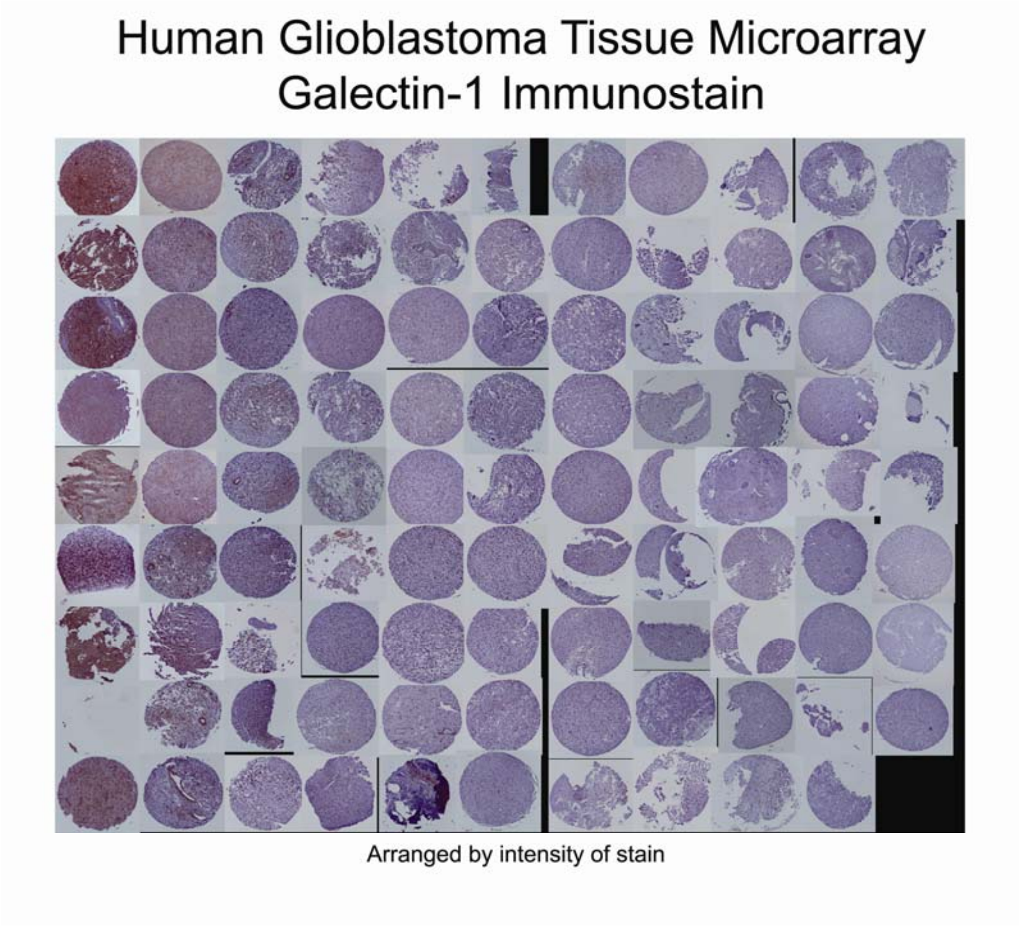

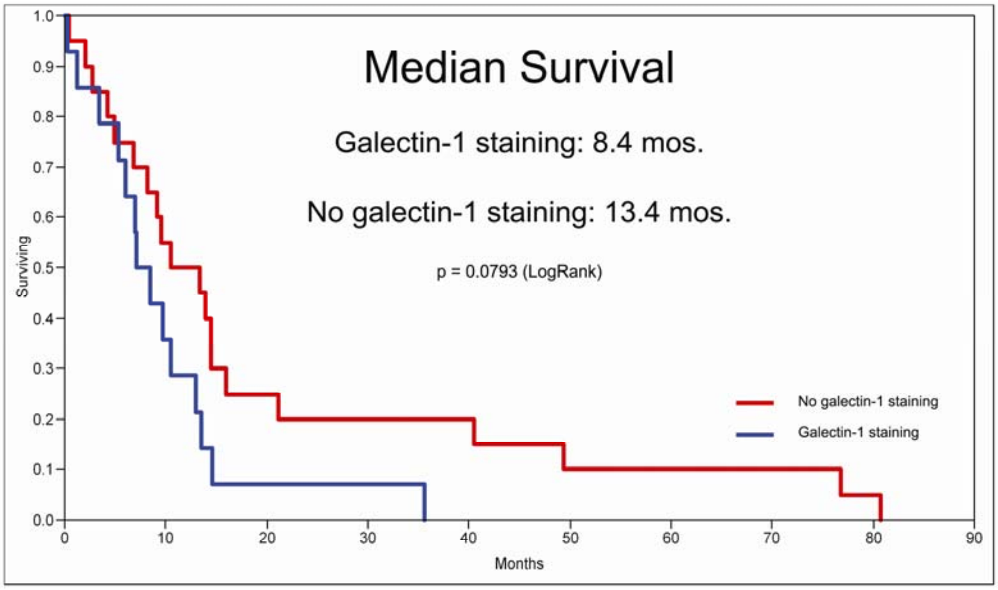

Supplemental Figure 1 . Galectin-1 staining correlates with patient survival. Using a tissue microarray created at Mayo Clinic, we stained glioblastoma samples from 34 separate patients using immunohistochemistry for galectin-1. A survival analysis revealed a trend towards shorter survival in those patients harboring galectin-1 positive tumors.

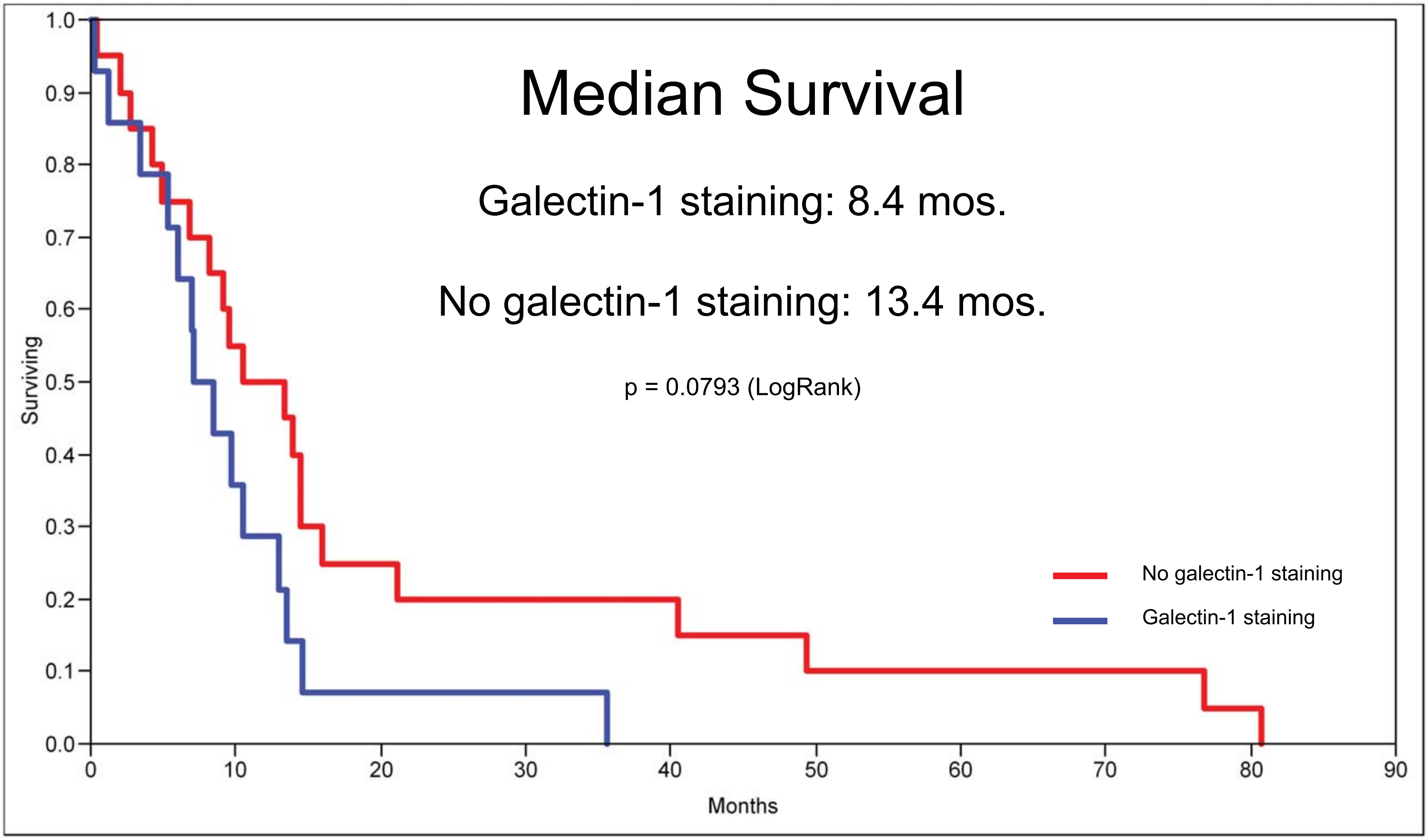
Median Survival
Galectin-1 staining: 8.4 mos.
No galectin-1 staining: 13.4 mos.
p = 0.0793 (LogRank)
No galectin-1 staining
Galectin-1 staining
Surviving
Months
1.0
0.9
0.8
0.7
0.6
0.5
0.4
0.3
0.2
0.1
0.0
0
10
20
30
40
50
60
70
80
90
